# Prevalence and profile of nocturnal disturbances in Chinese patients with advanced-stage Parkinson’s disease: a cross-sectional epidemiology study

**DOI:** 10.1186/s12883-021-02217-5

**Published:** 2021-05-12

**Authors:** Guiying He, Chun-Feng Liu, Qinyong Ye, Zhenguo Liu, Miao Jin, Huifang Shang, Ling Chen, Houzhen Tuo, Hong Jiang, Jifu Cai, Kalpesh Joshi, James Cooper, Lu Zi, Shengdi Chen

**Affiliations:** 1 Department of Neurology, Rui Jin Hospital Affiliated to Shanghai Jiao Tong University School of Medicine, Shanghai, China; 2 The Second Affiliated Hospital of Soochow University, Suzhou, China; 3 Fujian Medical University Union Hospital, Fuzhou, China; 4 Xin Hua Hospital Affiliated to Shanghai Jiao Tong University School of Medicine, Shanghai, China; 5 China-Japan Friendship Hospital, Beijing, China; 6 West China Hospital, Sichuan University, Chengdu, Sichuan China; 7 The First Affiliated Hospital of Sun Yat-sen University, Guangzhou, Guangdong China; 8 Beijing Friendship Hospital, Capital Medical University, Beijing, China; 9 Xiangya Hospital of Central South University, Changsha, Hunan China; 10 The University of Hong Kong-Shenzhen Hospital, Shenzhen, Guangdong China; 11 GSK, Mumbai, India; 12 GSK, Brentford, Middlesex UK; 13 GSK, Shanghai, China

**Keywords:** Parkinson’s disease, Nocturnal disturbances, Sleep disorders, Prevalence, Quality of life, China

## Abstract

**Background:**

The impact of nocturnal disturbance (ND) in Parkinson’s disease on quality of life of patients in Western Countries is increasingly understood. Our study aimed to investigate ND prevalence and its quality of life impact in patients with advanced Parkinson’s disease in China.

**Methods:**

In a multicenter, tertiary-care hospital, outpatient-based, cross-sectional study, patients with advanced Parkinson’s disease (Modified Hoehn & Yahr [H&Y] Stage II–IV with ≥3 h awake “off” time/day) from 10 tertiary hospitals throughout China completed the Parkinson’s Disease Sleep Scale-2 (PDSS-2) and Parkinson’s Disease Questionnaire-39 (PDQ-39). The primary endpoint was the percentage of patients with significant ND (PDSS-2 total score ≥ 15). Additional endpoints were demographic and clinical characteristics, PDSS-2 and PDQ-39 total and subscale scores, correlation between PDSS-2 and PDQ-39, and risk factors for ND and higher PDSS-2 or PDQ-39 scores.

**Results:**

Of 448 patients analyzed (mean age 63.5 years, 47.3% female), 70.92% (95% confidence interval: 66.71, 75.13) had significant ND. Presence of ND and higher PDSS-2 scores were associated with longer disease duration and higher H&Y stage. Presence of ND was also associated with more awake “off” time/day and female sex. PDQ-39 scores were significantly worse for patients with ND versus those without ND; worse scores were associated with more awake “off” time/day, female sex, and higher H&Y stage. PDSS-2 and PDQ-39 total scores were associated: Pearson correlation coefficient 0.62 (*p* < 0.001).

**Conclusions:**

In China, ND was highly prevalent in patients with advanced Parkinson’s disease and adversely impacted quality of life. This study highlights the importance of early diagnosis and optimized management of ND in patients with Parkinson’s disease in China.

**Supplementary Information:**

The online version contains supplementary material available at 10.1186/s12883-021-02217-5.

## Background

Parkinson’s disease (PD) is associated with nocturnal disturbance (ND) with prevalence ranging from 60 to 85% in Western countries [1, 2]. While the criteria for ND diagnosis have yet to be defined, the spectrum of symptoms of ND is wide and includes a number of PD-related motor symptoms (e.g., nocturnal and early morning akinesia/dystonia), treatment-related ND and/or psychiatric symptoms (e.g., hallucinations and vivid dreams), and other sleep disorders (e.g., insomnia) and, sleep-disordered breathing [including sleep apnea syndromes], restless legs syndrome [RLS], rapid eye movement sleep behavior disorder [RBD], excessive daytime sleepiness [EDS], and nocturia) [3, 4]. In patients with PD, sleep-related ND symptoms typically arise before onset of motor symptoms and worsen as disease progresses [5, 6]. This could be caused by progressive neurodegeneration affecting the circadian rhythm, the presence of motor complications and polytherapy, which can affect the sleep architecture [5–7]. As ND symptoms often lead to daytime complications like sleepiness, fatigue, and irritability, it can negatively impact a patient’s quality of life (QoL) [8]. Hence, it is imperative to continue to assess ND symptoms as the disease progresses to optimize clinical outcomes for patients with PD and improve QoL.

Assessment of ND is typically achieved by clinical review and screening instruments, such as the Parkinson’s Disease Sleep Scale (PDSS) [8, 9]. The impact of PD on QoL can be evaluated using instruments such as the PDQ-39 [10]. Both the PDSS and PDQ-39 have been validated in the Chinese language [11–13]. In 2011, the 2nd version of the PDSS (PDSS-2) was developed [14]; this has been validated in patients from Austria, Germany and the UK [15], and used in previous studies in Chinese patients [16, 17]; however, to our knowledge, the PDSS-2 has not been validated in the Chinese language to date.

Patients with PD experiencing significant ND usually have several characteristics in common. For example, in a hospital-based study in India, patients with PD and sleep problems had significantly worse disease, longer disease duration, higher levodopa dose, and higher total rigidity score than those without sleep disturbances [5]. Age and sex have not been associated with increased PDSS-2 score in previous studies [18–20]; however, both factors have been known to impact quality of life, and sex differences have been reported with respect to symptoms and risk for PD. [21–25] This suggests there may be several risk factors that are useful for identifying ND status in patients with PD.

In China, the prevalence of PD more than doubled from 1996 to 2016, and it was estimated that 1.4 million patients with PD were living in China in 2016 [26]. The recognition and management of non-motor symptoms in these patients, such as sleep disturbances, are evolving [27]. In 2018, the first Chinese consensus recommendations for the management of sleep disturbances in patients with PD were published [4]. While these recommendations were helpful, they did not address the wider concept of ND in patients with PD, particularly related to motor and treatment-related symptoms, and their optimal management warrants further consideration.

This study aimed to quantify the prevalence of ND in patients with advanced-stage PD, determine the profile of patients with ND, and investigate the impact of ND on QoL for the first time in China using the Chinese versions of the Parkinson’s Disease Sleep Scale-2 (PDSS-2) and the Parkinson’s Disease Questionnaire (PDQ-39). As regional differences in China, such as ethnicity and healthcare access, could potentially impact the findings [28, 29], we included patients with PD from 10 different outpatient departments in tertiary hospitals.

## Methods

### Study design

This was a multicenter, tertiary-care hospital, outpatient-based, cross-sectional study conducted across 10 sites throughout China to represent a broad Chinese population. The trial was conducted in accordance with the Declaration of Helsinki and approved by the Research Ethics Committee of Rui Jin Hospital affiliated to Shanghai Jiao Tong University. After obtaining informed consent, patients with PD were recruited at routine hospital visits and evaluated during a single study visit. Two questionnaires were given to the patients to complete: PDSS-2 and PDQ-39 [10, 14]. Patients completed the questionnaires themselves; however, investigators could help when the patient had writing difficulty due to severe motor symptoms. The investigators also performed a physical examination, determined the Modified Hoehn and Yahr (H&Y) stage, and collected information on demographics and medical history. Eligible patients were consecutively invited to participate to avoid selection bias.

### Study population

Sample size was based primarily on feasibility since no formal hypothesis was tested in this study. A target of 450 patients with advanced-stage PD were to be recruited under the assumption of a 60% prevalence of ND in populations with PD, providing a precision of 4.5% in prevalence estimate.

Inclusion criteria for the study were ≥ 30 years of age, diagnosed with idiopathic PD according to the UK Parkinson’s Disease Society Brain Bank criteria, Stage II to IV of the Modified H&Y scale, wearing off (motor fluctuations) despite optimized pharmacological treatment (levodopa monotherapy or adjunctive therapies), history of ≥3 h awake “off” time per day for the past 4 weeks based on the investigator’s knowledge of the patient, able to comprehend Mandarin Chinese, and provide written informed consent. Exclusion criteria were clinical diagnosis of dementia, atypical Parkinsonism, unable to complete interviews as assessed by investigators, unable to complete the PDSS-2 and PDQ-39 with the support of the investigator, or currently participating in any PD clinical studies testing interventional drugs or disease management programs.

### Study endpoints and statistical analyses

The primary endpoint of the study was percentage of patients with advanced-stage PD with evidence of significant ND. ND was defined as a PDSS-2 total score ≥ 15 [30]. Secondary endpoints included demographic and clinical characteristics, total score and subscale scores of PDSS-2 (disturbed sleep, motor symptoms at night, and PD symptoms at night), total score and subscale scores of PDQ-39 (mobility, activities of daily living, emotional wellbeing, stigma, social support, cognitions, communication, and bodily discomfort) of advanced-stage PD patients with ND, and the correlation between the PDSS-2 total scores and subscale scores with PDQ-39 total scores. For both outcome measures, higher scores indicated worse outcomes.

Analyses were performed after all patients had completed the study and after database freeze on the all analysis set, which contained patients who passed screening and had ≥1 item assessment of PDSS-2 or PDQ-39 and did not have any important protocol deviations that significantly impacted the completeness, accuracy, and/or reliability of the trial data or significantly affected a patient’s rights, safety, or wellbeing.

The proportion of patients with PD with ND was estimated by observed percentage with 95% confidence interval (CI). Descriptive statistics were used to analyze demographic and clinical characteristics and total and subscale scores of PDSS-2 and PDQ-39. For comparisons between continuous variables with normal data distribution, the *t*-test was used. The Wilcoxon rank sum test was used for analyses of continuous variables with non-normal data distributions. Categorical variables were analyzed using the Chi-square test and where there were lower than expected frequencies (< 5), the variables were analyzed with Fisher’s exact test. Risk factors for ND status were assessed by a multivariate logistic model with age, sex, disease duration, disease stage, average time of awake “off” time included as fixed factors. Significance of any differences between PDSS-2 subscale scores was determined using the Analysis of Covariance model with ND status, sex, and disease stage as fixed factors, and age and disease duration as continuous covariates. Linear regression of PDSS-2 scores included sex and disease stage as fixed factors, and age and disease duration as continuous explanatory variables. A linear regression of PDQ-39 total score was performed with sex and disease stage of PD included as fixed factors and PDSS-2 total score, age, disease duration included as continuous explanatory variables. The correlation between the PDSS-2 total and subscale scores with the PDQ-39 total score was analyzed using Pearson’s correlation.

## Results

### Patients

Patients were recruited from 10 sites from different geographic regions in China during the study period (December 27, 2018–May 8, 2019) (Supplementary figure 1). The study screened 453 patients and enrolled 450 patients. The all analysis set contained 448 patients, representing 99.6% of the 453 patients who provided informed consent (Supplementary figure 2). Two patients were excluded from the all analysis set as they did not meet the inclusion criteria.

### Demographics and clinical characteristics

Patients had a mean age of 63.5 years, and 52.7% were male in the all analysis set (Table 1). Nearly all patients were of Chinese heritage (99.8%). A single patient had Japanese heritage. Of the 447 patients with PDSS total score results, 70.92% (95% CI: 66.71, 75.13) of patients had significant ND. Patient demographics were similar between the groups of patients with and without significant ND, but significant differences were observed in their clinical characteristics (Table 1). Compared with patients without ND, those with ND had a significantly longer mean disease duration (7.41 vs 6.00 years, *p* = 0.003), increased mean awake “off” time/day (5.51 vs 4.64 h, *p* = 0.001), and more advanced H&Y stage (*p* < 0.001).

**Table 1:** Patient demographic and clinical characteristics

	With ND (***n*** = 317)	Without ND (***n*** = 130)	Total^**a**^ (***N*** = 448)
**Age (years)**			
Mean (SD)	63.2 (9.38)	64.1 (9.45)	63.5 (9.39)
p-value^b^		0.385^c^	
**Sex, n (%)**			
Male	160 (50.5%)	76 (58.5%)	236 (52.7%)
Female	157 (49.5%)	54 (41.5%)	212 (47.3%)
p-value^b^		0.124^d^	
**Disease duration (years)**			
Mean (SD)	7.41 (4.281)	6.00 (4.904)	7.00 (4.506)
p-value^b^		0.003^c^	
**Average time of awake “off” per day (hours)**			
Mean (SD)	5.51 (2.807)	4.64 (2.386)	5.27 (2.720)
p-value^b^		0.001^c^	
**Disease stage (H&Y), n (%)**			
Stage II	90 (28.4)	63 (48.5)	153 (34.2)
Stage IIS	92 (29.0)	28 (21.5)	120 (26.8)
Stage III	109 (34.4)	35 (26.9)	145 (32.4)
Stage IV	26 (8.2)	4 (3.1)	30 (6.7)
p-value^b^		< 0.001^d^	

^a^A single patient was excluded in both of with/without ND group due to missing one item of PDSS-2 score; ^b^comparison between with and without ND; ^c^determined using *t*-test; ^d^determined using Pearson Chi-square test

*H&Y* Modified Hoehn & Yahr, *ND* Nocturnal disturbance, Parkinson’s Disease Sleep Scale 2nd version, *SD* Standard deviation

The following clinical characteristics were identified as risk factors for ND in patients with PD: disease duration (odds ratio; 95%CI] for 1 year of disease duration increase = 1.06 [1.01, 1.12]; *p* = 0.022), and time awake “off” per day (for 1 h “off” time increase = 1.12 [1.02, 1.23]; *p* = 0.015), disease stage (for 1 disease stage increase = 1.36 [1.06, 1.74]; *p* = 0.014).

### PDSS-2 score

**Fig. 1:**
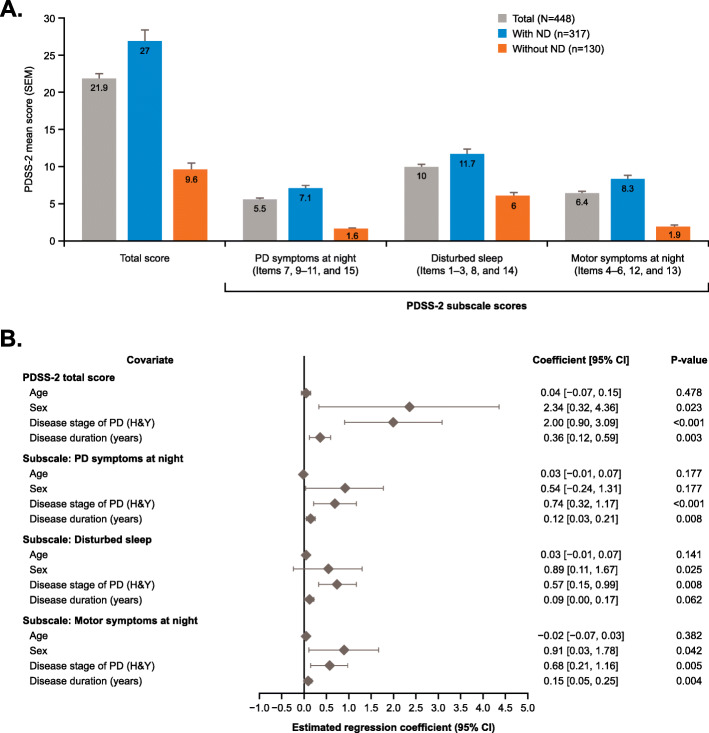
Mean PDSS-2 total and subscale scores (**a**) and regression analysis (**b**) for patients with advanced-stage PD with and without ND (all analysis set). For part **a**, statistical analysis was not performed for the PDSS-2 total score. For part **b**, the estimated regression coefficient for age, H&Y stage (Stage II = 1, Stage IIS = 2, Stage III = 3, Stage IV = 4) and disease duration are calculated to compare the pairwise difference in 1 higher unit change. The estimated regression coefficient of sex is calculated with male as the reference level; *p < 0.001. CI, confidence interval; H&Y, Modified Hoehn & Yahr; ND, nocturnal disturbance; PDSS-2, Parkinson’s Disease Sleep Scale 2nd version; PD, Parkinson’s disease; SEM, standard error of the mean

Mean PDSS-2 total scores for patients with ND (PDSS-2 ≥ 15) and without ND (PDSS-2 < 15) were 27.0 and 9.6, respectively (Fig. 1). Subscale scores (PD symptoms at night, disturbed sleep, and motor symptoms at night) were significantly different between the two groups (*p* < 0.001).

Female sex (*p* = 0.023), higher H&Y stage (*p* < 0.001), and longer disease duration (*p* = 0.003) were associated with a greater probability of a higher PDSS-2 total score (Fig. 1b) as determined by linear regression analysis. The PDSS-2 subscale scores were also associated with higher H&Y stages (all 3 subscales; *p* < 0.01). Longer duration of disease was associated with higher motor symptoms and PD symptoms at night subscale scores (both *p* < 0.01), and female sex was associated with higher motor symptoms at night and disturbed sleep subscale scores (both *p* < 0.05). Individual item scores were all significantly different (*p* < 0.001) between patients with and without ND (Supplementary table1).

### PDQ-39 score and correlation between PDSS-2 scores and PDQ-39 scores

**Fig. 2:**
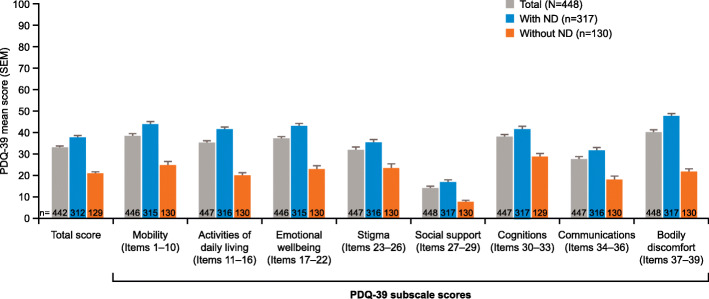
PDQ-39 total and subscale scores for patients with advanced-stage PD with and without ND (all analysis set). *p < 0.001 (with vs without ND). ND, nocturnal disturbance; PD, Parkinson’s disease; PDQ-39, Parkinson’s Disease Questionnaire-39; SEM, standard error of the mean

**Fig. 3:**
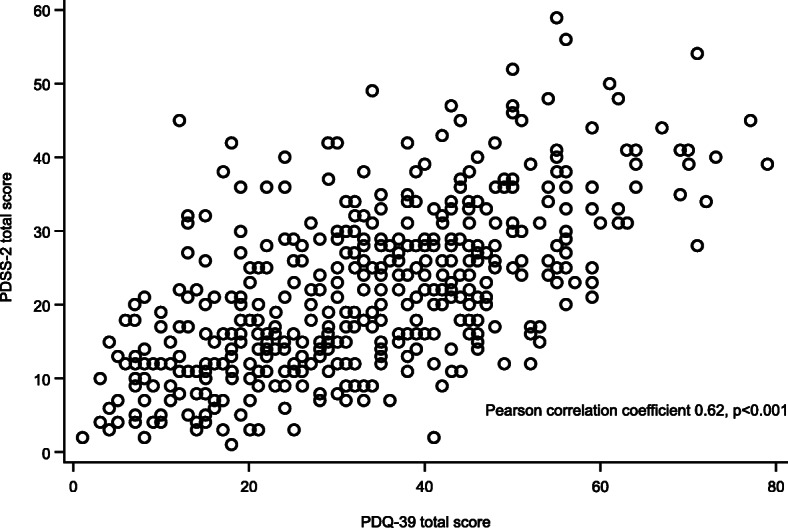
Scatter plot for PDSS-2 total score versus PDQ-39 total score (all analysis set). Includes *N* = 442 patients. PDSS-2, Parkinson’s Disease Sleep Scale 2nd version; PDQ-39, Parkinson’s Disease Questionnaire-39

The mean PDQ-39 total (37.8 vs 20.8) and subscale scores for patients with significant ND were significantly higher (*p* < 0.001) than for patients without ND (Fig. 2). Pearson correlation analysis for PDSS-2 total score and PDQ-39 total score showed a significant association between the two assessment scores (estimated regression coefficient = 0.62, *p* < 0.001) (Fig. 3). Likewise, the Pearson correlation analyses for the PDSS-2 subscale scores (PD symptoms at night, disturbed sleep, and motor symptoms at night) and the PDQ-39 total score also showed significant correlations (coefficient = 0.56, 0.43, and 0.56, respectively; *p* < 0.001 for each correlation).

A multivariate linear regression analysis demonstrated associations between ND status and PDQ-39 total score after controlling for the following factors: age, sex, disease duration, H&Y stage, and awake “off” time/day. A status shift from without significant ND to with significant ND was associated with a significant mean increase of 13.83 (95% CI: 11.08, 16.59) in the PDQ-39 score (*p* < 0.001). In addition, female sex (*p* = 0.029), higher H&Y stage (*p* < 0.001), and more awake “off” time/day (*p* < 0.001) were also associated with a significantly worse QoL as measured by the PDQ-39 in the all analysis set (Supplementary table 2).

## Discussion

This study found that 71% of patients with advanced PD recruited from different regions of China experienced significant ND. This is consistent with previously reported prevalence observations in China (48–89%) [4, 31], and in Western countries (60%) [1]. In total, 448 patients from tertiary hospitals at 10 different sites were evaluated during the study. The geographic locations of these 10 sites spanned most areas of China.

We found that increased disease severity, longer PD duration, and more mean awake “off” time/day were risk factors for significant ND. While these data indicate that the most-affected patients with PD are at greatest risk of ND, they also suggest that poorly managed motor dysfunction may be related to ND in some patients. Optimizing medication to ensure continuous dopaminergic exposure throughout a 24-h period with the aim of reducing nocturnal “off” time may be effective at improving sleep within these patients. This may be achieved by using dopamine agonists, catechol-O-methyltransferase inhibitors, monoamine oxidase B inhibitors, or controlled-release levodopa formulations to cover night periods and to promote continuous dopaminergic receptor stimulation, as suggested in local guidelines [4, 32].

To evaluate ND in patients with PD, the PDSS-2 was employed. The PDSS-2 is designed to assess sleep disorders and other nocturnal symptoms in patients with PD and is an easy, valid, and reliable scale, which may be useful for measuring treatment responses [14]. Moreover, it has been found to correlate positively with polysomnographic parameters [33]. In this study, the cutoff of total score ≥ 15 was employed to identify significant ND. This cutoff was previously determined in patients with PD in Japan in a cross-sectional study and was found to provide 72.1% sensitivity and 72.9% specificity in distinguishing poor sleepers [30]. In our population of patients with PD, this cutoff was associated with longer disease duration and higher H&Y stages: characteristics previously associated with sleep problems in PD. [5] Furthermore, the mean PDSS-2 total score for patients with significant ND was + 17.4 points higher than those without significant ND, a difference far greater than the previously reported minimal clinically important difference of + 2.07 points for worsening of PDSS-2 [34]. Taken together, this suggests that the population identified was experiencing significant ND.

Higher PDSS-2 scores were associated with female sex, higher H&Y stages, and longer disease duration. The strongest associations were found with female sex and an increase in H&Y stage (estimated regression coefficients: 2.34 and 2.00, respectively). Increased age was not significantly associated with worse PDSS-2 total scores, which is consistent with previous reports [18–20]. This suggests that the increased lifespan of women [35] was not the reason for the observed sex effect. While previous studies did not find an association between female sex and PDSS-2 score [18–20], differences in the development and phenotypical expression between male and female patients with PD suggest that such a relationship is plausible [21]. Furthermore, a cross-sectional analysis of drug-naïve patients with PD did find that sleep-related non-motor symptoms were more frequent in females compared to males [23]. However, further research will be required to understand the sex differences in ND for patients with PD. Increased disease severity was associated with higher scores in all subscales of the PDSS-2, an effect which has been demonstrated previously for total PDSS-2 score [18, 19]. Longer disease duration was also associated with higher PDSS-2 score, which has also been observed previously [18]. The subscales of PDSS-2 were also associated with several patient characteristics, for instance, female sex was associated with increased subscale scores of disturbed sleep and motor symptoms at night.

The PDQ-39 is a validated, reliable scale for measuring QoL in patients with Parkinson’s disease that has been validated in the Chinese language [11, 12]. Using this measure, it was found that patients with significant ND had significantly worse QoL than patients without ND for all domains tested with a mean difference of + 17.0 points. This difference is far above the reported minimal clinically important difference of + 4.22 for worsening of the PDQ-39 [36]. Using multivariate linear regression analysis, it was determined that the presence of ND is associated with an increase in PDQ-39 total score of 13.83 points after controlling other factors, like age, sex, disease duration, H&Y stage, and awake “off” time/day. This increase is much higher than those found for the other clinical factors found to be significantly associated with increases in PDQ-39 total scores, suggesting that ND are particularly detrimental to QoL in these patients. Given the significant positive correlation between PDSS-2 total scores and subscales scores with PDQ-39 total scores, these data indicate that ND has a significant negative impact on patient QoL and should be an important consideration for clinicians treating patients with PD in China and the wider world.

A similar multicenter, observational study (Zhang et al.), published recently, detailed investigations of sleeping disorders in patients with PD from Shanghai hospitals [31]. The study corroborates many of our own findings. Specifically, it reports that the number of sleeping disorders a patient has correlates with impaired QoL. This supports our finding that the severity of NDs is associated with worse QoL. While our study appears quite similar to Zhang et al., we did note that our patient population had a longer mean PD duration compared with patients included in Zhang et al. (7 vs 5.5 years).

An important strength of this study is the use of validated, PD-specific instruments for measuring sleep disturbance (PDSS-2) and QoL (PDQ-39) in PD. A further strength was the inclusion of tertiary hospitals in multiple regions of China. Tertiary hospitals in China have more experience with PD than primary or secondary hospitals and are preferred by patients [28]. Studying multiple regions in China not only allows patients of different ethnic populations to be included; it also limits effects on the data caused by regional differences in healthcare [28, 29]. This regional variety was lacking in the previously discussed Zhang et al. study of sleeping disorders in PD patients [31]. This study focused exclusively on patients from Shanghai hospitals. Weaknesses of the present study include a lack of longitudinal data. Our study presents data from a single interview. Multiple interviews would provide a broader view of each patient and give an indication of how the two measures (PDSS-2 and PDQ-39) change over time. Additionally, we did not stratify data by treatment type, while the current study gives an overall view of current clinical practice and suggests gaps in treatment, it does not allow conclusions to be made about the effects of optimized therapy. Finally, the inclusion criteria used the UK Parkinson’s Disease Society Brain Bank criteria for the diagnosis of idiopathic PD, whereas the Movement Disorder Society PD diagnostic criteria have become the gold standard in clinical practice and research settings [37], and have been recommended for use in clinical work in China [38]. The results should be considered in light of the differences in diagnostic criteria.

## Conclusions

These results both confirm that a significant portion of patients with advanced PD in China experience ND and highlight how debilitating ND is to patients. They also should give clinicians confidence that the PDSS-2, a simple bedside assessment tool with a good correlation to polysomnography, can be easily implemented in clinical practice to identify patients experiencing ND. Given the relatively simple identification of ND in patients with PD and the potential to treat these patients (at least in part) using optimized medication for PD motor symptoms, it is important for clinicians to routinely consider the best management of their patients in this context.

## Supplementary Information

**Additional file 1 **Study 207,944 Nocturnal disturbances. **Figure S1.** Study sites. **Figure S2.** Patient disposition. **Table S1.** Item scores for PDSS-2. **Table S2.** Linear regression of PDQ-39 total score.

## Data Availability

Anonymized individual participant data and study documents can be requested for further research from www.clinicalstudydatarequest.com.

## Abbreviations

CI: Confidence interval
GSK: GlaxoSmithKline
H&Y: Modified Hoehn & Yahr
ND: Nocturnal disturbance
PD: Parkinson’s disease
PDSS: Parkinson’s Disease Sleep Scale
PDSS-2: Parkinson’s Disease Sleep Scale-2
PDQ-39: Parkinson’s Disease Questionnaire-39
QoL: Quality of life
SD: Standard deviation
SEM: Standard error of the mean

## References

[1] Grandas F, Iranzo A (2004). Nocturnal problems occurring in Parkinson’s disease. Neurology..

[2] Hermanowicz N, Jones SA, Hauser RA (2019). Impact of non-motor symptoms in Parkinson's disease: a PMDAlliance survey. Neuropsychiatr Dis Treat.

[3] Barone P, Amboni M, Vitale C, Bonavita V (2004). Treatment of nocturnal disturbances and excessive daytime sleepiness in Parkinson's disease. Neurology..

[4] Liu CF, Wang T, Zhan SQ, Geng DQ, Wang J, Liu J (2018). Management recommendations on sleep disturbance of patients with Parkinson's disease. Chin Med J.

[5] Kumar S, Bhatia M, Behari M (2002). Sleep disorders in Parkinson’s disease. Mov Disord.

[6] Politis M, Wu K, Molloy S, Bain PG, Chaudhuri KR, Piccini P (2010). Parkinson’s disease symptoms: the patient’s perspective. Mov Disord.

[7] Willison LD, Kudo T, Loh DH, Kuljis D, Colwell CS (2013). Circadian dysfunction may be a key component of the non-motor symptoms of Parkinson's disease: insights from a transgenic mouse model. Exp Neurol.

[8] Chaudhuri KR, Pal S, DiMarco A, Whately-Smith C, Bridgman K, Mathew R (2002). The Parkinson’s disease sleep scale: a new instrument for assessing sleep and nocturnal disability in Parkinson's disease. J Neurol Neurosurg Psychiatry.

[9] Hogl B, Arnulf I, Comella C, Ferreira J, Iranzo A, Tilley B (2010). Scales to assess sleep impairment in Parkinson's disease: critique and recommendations. Mov Disord.

[10] Peto V, Jenkinson C, Fitzpatrick R, Greenhall R (1995). The development and validation of a short measure of functioning and well being for individuals with Parkinson’s disease. Qual Life Res.

[11] Tsang KL, Chi I, Ho SL, Lou VW, Lee TM, Chu LW (2002). Translation and validation of the standard Chinese version of PDQ-39: a quality-of-life measure for patients with Parkinson’s disease. Mov Disord.

[12] Luo W, Gui XH, Wang B, Zhang WY, Ouyang ZY, Guo Y (2010). Validity and reliability testing of the Chinese (mainland) version of the 39-item Parkinson's disease questionnaire (PDQ-39). J Zhejiang Univ Sci B.

[13] Wang G, Cheng Q, Zeng J, Bai L, Liu GD, Zhang Y (2008). Sleep disorders in Chinese patients with Parkinson's disease: validation study of a Chinese version of Parkinson’s disease sleep scale. J Neurol Sci.

[14] Trenkwalder C, Kohnen R, Hogl B, Metta V, Sixel-Doring F, Frauscher B (2011). Parkinson’s disease sleep scale--validation of the revised version PDSS-2. Mov Disord.

[15] Trenkwalder C, Kies B, Rudzinska M, Fine J, Nikl J, Honczarenko K (2011). Rotigotine effects on early morning motor function and sleep in Parkinson's disease: a double-blind, randomized, placebo-controlled study (RECOVER). Mov Disord.

[16] Liu H, Ou R, Wei Q, Hou Y, Cao B, Zhao B (2019). Rapid eye movement behavior disorder in drug-naive patients with Parkinson's disease. J Clin Neurosci.

[17] Zhang ZX, Liu CF, Tao EX, Shao M, Liu YM, Wang J (2017). Rotigotine transdermal patch in Chinese patients with advanced Parkinson's disease: a randomized, double-blind, placebo-controlled pivotal study. Parkinsonism Relat Disord.

[18] Chang CW, Fan JY, Chang BL, Wu YR (2019). Anxiety and levodopa equivalent daily dose are potential predictors of sleep quality in patients with Parkinson disease in Taiwan. Front Neurol.

[19] Martinez-Martin P, Wetmore JB, Rodríguez-Blázquez C, Arakaki T, Bernal O, Campos-Arillo V (2019). The Parkinson's disease sleep Scale-2 (PDSS-2): validation of the Spanish version and its relationship with a roommate-based version. Mov Disord Clin Pract.

[20] Melka D, Tafesse A, Bower JH, Assefa D (2019). Prevalence of sleep disorders in Parkinson’s disease patients in two neurology referral hospitals in Ethiopia. BMC Neurol.

[21] Cerri S, Mus L, Blandini F (2019). Parkinson’s disease in women and men: What’s the difference?. J Parkinsons Dis.

[22] Chen K, Yang YJ, Liu FT, Li DK, Bu LL, Yang K (2017). Evaluation of PDQ-8 and its relationship with PDQ-39 in China: a three-year longitudinal study. Health Qual Life Outcomes.

[23] Hu T, Ou R, Liu H, Hou Y, Wei Q, Song W (2018). Gender and onset age related-differences of non-motor symptoms and quality of life in drug-naïve Parkinson's disease. Clin Neurol Neurosurg.

[24] Wooten GF, Currie LJ, Bovbjerg VE, Lee JK, Patrie J (2004). Are men at greater risk for Parkinson's disease than women?. J Neurol Neurosurg Psychiatry.

[25] Ma CL, Su L, Xie JJ, Long JX, Wu P, Gu L (2014). The prevalence and incidence of Parkinson's disease in China: a systematic review and meta-analysis. J Neural Transm (Vienna).

[26] G. B. D. Parkinson’s Disease Collaborators (2018). Global, regional, and national burden of Parkinson’s disease, 1990–2016: a systematic analysis for the Global Burden of Disease Study 2016. Lancet Neurol.

[27] Shen Y, Liu CF (2018). Sleep disorders in Parkinson's disease: present status and future prospects. Chin Med J.

[28] Li G, Ma J, Cui S, He Y, Xiao Q, Liu J (2019). Parkinson's disease in China: a forty-year growing track of bedside work. Transl Neurodegener.

[29] Wang T, Zeng R (2015). Addressing inequalities in China's health service. Lancet.

[30] Suzuki K, Miyamoto T, Miyamoto M, Suzuki S, Numao A, Watanabe Y (2015). Evaluation of cutoff scores for the Parkinson's disease sleep scale-2. Acta Neurol Scand.

[31] Zhang Y, Zhao JH, Huang DY, Chen W, Yuan CX, Jin LR (2020). Multiple comorbid sleep disorders adversely affect quality of life in Parkinson’s disease patients. NPJ Parkinsons Dis.

[32] Chen S, Chan P, Sun S, Chen H, Zhang B, Le W (2016). The recommendations of Chinese Parkinson’s disease and movement disorder society consensus on therapeutic management of Parkinson’s disease. Transl Neurodegener.

[33] Jasti DB, Mallipeddi S, Apparao A, Vengamma B, Kolli S, Mohan A (2018). Quality of sleep and sleep disorders in patients with parkinsonism: a polysomnography based study from rural South India. J Neurosci Rural Pract.

[34] Horvath K, Aschermann Z, Acs P, Deli G, Janszky J, Komoly S (2015). Minimal clinically important difference on Parkinson’s disease sleep scale 2nd version. Parkinsons Dis.

[35] Le Y, Ren J, Shen J, Li T, Zhang CF (2015). The changing gender differences in life expectancy in Chinese cities 2005–2010. PLoS One.

[36] Horvath K, Aschermann Z, Kovacs M, Makkos A, Harmat M, Janszky J (2017). Changes in quality of life in Parkinson’s disease: how large must they be to be relevant?. Neuroepidemiology..

[37] Postuma RB, Berg D, Stern M, Poewe W, Olanow CW, Oertel W (2015). MDS clinical diagnostic criteria for Parkinson's disease. Mov Disord.

[38] Li J, Jin M, Wang L, Qin B, Wang K (2017). MDS clinical diagnostic criteria for Parkinson’s disease in China. J Neurol.

