# Supplementary material for: Prevalence and profile of nocturnal disturbances in Chinese patients with advanced-stage Parkinson’s disease: a cross-sectional epidemiology study
Source: BMC Neurol. 2021 May 12;21:194. doi: 10.1186/s12883-021-02217-5 (PMC8114718; doi:10.1186/s12883-021-02217-5)
Supplement: Supplementary file 1 — Additional file 1 Study 207,944 Nocturnal disturbances. Figure S1. Study sites. Figure S2. Patient disposition. Table S1. Item scores for PDSS-2. Table S2. Linear regression of PDQ-39 total score. [file 12883_2021_2217_MOESM1_ESM.docx]

Supplementary tables and figures for:

Prevalence and profile of nocturnal disturbances in Chinese patients with advanced-stage Parkinson’s disease: a cross-sectional epidemiology study

Guiying He et al.

Corresponding author:

Shengdi Chen
Address: Department of Neurology, Rui Jin Hospital Affiliated to Shanghai Jiao Tong University School of Medicine, Shanghai 200025, China
Tel: +86 21-6445-4473
Email: chensd@rjh.com.cn

## Supplementary Figure 1. Study sites


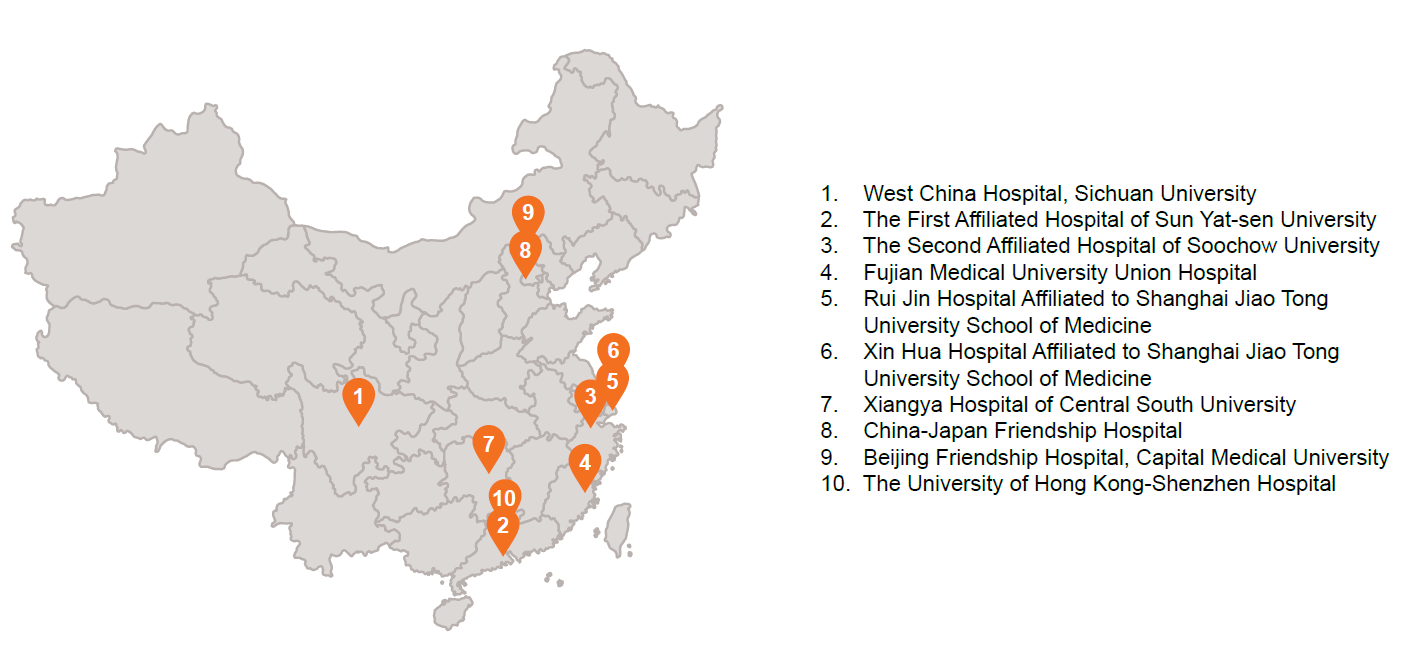


## Supplementary Figure 2. Patient disposition


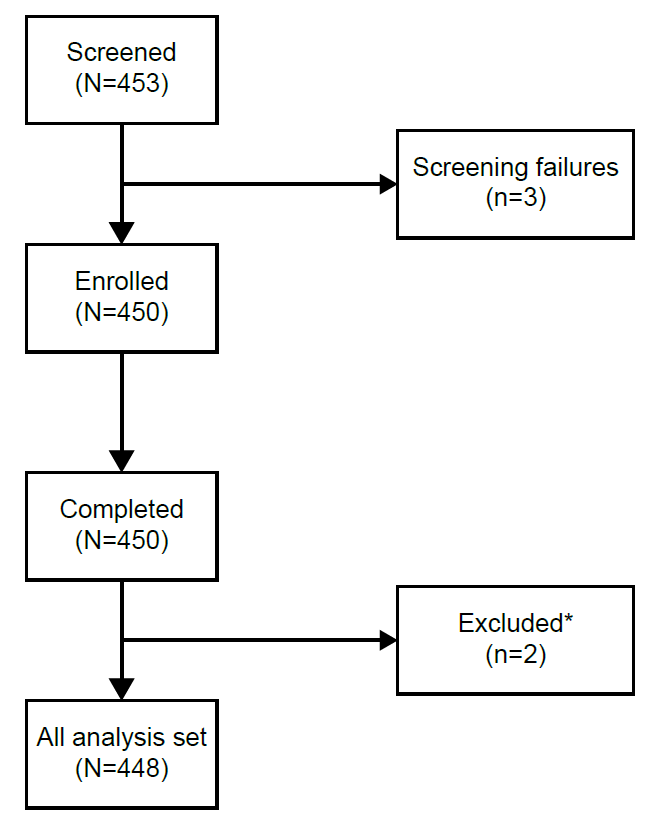


*Two patients were excluded from the all analysis set for not meeting inclusion criteria for analysis. These patients did not use levodopa monotherapy or adjunctive therapy before enrolment to the study.

## Supplementary Table 1. Item scores for PDSS-2

|  | **With ND  (n=317)** | **Without ND (n=130)** | **Total  (N=448)** |
| --- | --- | --- | --- |
| **Overall, did you sleep well?** |  |  |  |
| Median (min., max.) | 2 (0, 4) | 1 (0, 4) | 2 (0, 4) |
| p-value |  |  | p<0.001 |
| **Difficulty falling asleep each night** |  |  |  |
| Median (min., max.) | 2 (0, 4) | 0 (0, 4) | 1 (0, 4) |
| p-value |  |  | p<0.001 |
| **Difficulty staying asleep** |  |  |  |
| Median (min., max.) | 3 (0, 4) | 1 (0, 4) | 3 (0, 4) |
| p-value |  |  | p<0.001 |
| **Restlessness leg/arm disrupt sleep** |  |  |  |
| Median (min., max.) | 2 (0, 4) | 0 (0, 4) | 1 (0, 4) |
| p-value |  |  | p<0.001 |
| **Urge to move leg/arm disrupt sleep** |  |  |  |
| Median (min., max.) | 2 (0, 4) | 0 (0, 4) | 1 (0, 4) |
| p-value |  |  | p<0.001 |
| **Distressing dreams at night** |  |  |  |
| Median (min., max.) | 2 (0, 4) | 0 (0, 4) | 1 (0, 4) |
| p-value |  |  | p<0.001 |
| **Distressing hallucination at night** |  |  |  |
| Median (min., max.) | 0 (0, 4) | 0 (0, 3) | 0 (0, 4) |
| p-value |  |  | p<0.001 |
| **Get up at night to pass urine** |  |  |  |
| Median (min., max.) | 4 (0, 4) | 3 (0, 4) | 4 (0, 4) |
| p-value |  |  | p<0.001 |
| **Uncomfortable due to immobility** |  |  |  |
| Median (min., max.) | 3 (0, 4) | 0 (0, 4) | 2 (0, 4) |
| p-value |  |  | p<0.001 |
| **Pain arms/legs woke you** |  |  |  |
| Median (min., max.) | 1 (0, 4) | 0 (0, 2) | 0 (0, 4) |
| p-value |  |  | p<0.001 |
| **Muscle cramps arm/leg woke you** |  |  |  |
| Median (min., max.) | 1 (0, 4) | 0 (0, 2) | 1 (0, 4) |
| p-value |  |  | p<0.001 |
| **Wake early-painful posture arm/leg** |  |  |  |
| Median (min., max.) | 1 (0, 4) | 0 (0, 2) | 0 (0, 4) |
| p-value |  |  | p<0.001 |
| **On waking, experience tremor** |  |  |  |
| Median (min., max.) | 1 (0, 4) | 0 (0, 4) | 0 (0, 4) |
| p-value |  |  | p<0.001 |
| **Feel tired/sleepy after waking** |  |  |  |
| Median (min., max.) | 2 (0, 4) | 0 (0, 4) | 1 (0, 4) |
| p-value |  |  | p<0.001 |
| **Wake up-snore/difficult breathing** |  |  |  |
| Median (min., max.) | 0 (0, 4) | 0 (0, 4) | 0 (0, 4) |
| p-value |  |  | p<0.001 |

A single patient was excluded in both of with/without ND group due to missing one item of PDSS-2 score. The ANCOVA model was used for the analysis of individual PDSS-2 item scores with ND status, sex (female vs male), and H&Y stage (Stage IV vs Stage III vs Stage IIS vs Stage II) included as fixed factors, and age, disease duration as continuous covariates. ANCOVA, analysis of covariance; H&Y, Modified Hoehn & Yahr; max., maximum; min., minimum; ND, nocturnal disturbance; PD, Parkinson’s disease; PDSS-2, Parkinson’s Disease Sleep Scale 2nd version.

## Supplementary Table 2. Linear regression of PDQ-39 total score

| **Covariate** | **Estimated regression coefficient (95% CI)** | **p-value** |
| --- | --- | --- |
| **ND status** | 13.83 (11.08, 16.59) | <0.001 |
| **Age** | -0.07 (-0.20, 0.06) | 0.308 |
| **Sex** | 2.73 (0.27, 5.18) | 0.029 |
| **Disease stage of PD** | 4.30 (2.93, 5.68) | <0.001 |
| **Disease duration, years** | 0.22 (-0.07, 0.51) | 0.143 |
| **Average time of awake “off” per day, hours** | 0.91 (0.45, 1.37) | <0.001 |

The impact of the ND status on the PDQ-39 total score was assessed by a multivariate linear model with age, sex, disease duration, disease stage, average time of awake time adjusted. The estimated regression coefficient for age, H&Y stage (Stage II = 1, Stage IIS = 2, Stage III = 3, Stage IV = 4), disease duration, and average time of awake time are calculated to compare the pairwise difference in 1 higher unit change. The estimated regression coefficient of sex is calculated with male as the reference level. The estimated regression coefficient of ND status is calculated with without ND as the reference level.

CI, confidence interval; H&Y, Modified Hoehn & Yahr; ND, nocturnal disturbance; PD, Parkinson’s disease; PDQ-39, Parkinson’s Disease Questionnaire-39.
